# Thank you to *eClinical Medicine's* clinical and statistical peer reviewers in 2022

**DOI:** 10.1016/j.eclinm.2023.101843

**Published:** 2023-01-23

Peter Aaby

Jeremiah Aakre

Julie Aarestrup

Daniel Aaron

Faisal Abbas

Enoch Abbey

Siddig Abdallah

Nagla Abdel Karim

Christina Abdel Shaheed

Mona Abdelrahman

Jamshid Abdul-Ghafar

Samia Abdul-Rahman

Zainab Abdulrasol

Takumi Abe

Behnoush Abedi-Ardekani

Ramy Abou Ghayda

Barham Abu Dayyeh

Niveen Abu-Rmeileh

Maurizio Acampa

Giovanni Adami

Venkateswar Addala

Yaw Addo

Adeyinka Adegbosin

Brian Adkins

Qorinah Estiningtyas Sakilah Adnani

Douglas Adu-Fokuo

Bamgboye Afolabi

Shoaib Afzal

Wonder Agbemavi

Liliana Aguayo-Markes

Suleiman Idris Ahmad

Syed Uzair Ahmed

Mark Ainsworth

Tanimola M Akande

Samuel Akech

Onno Akkerman

Gulali Aktas

Taghreed Al Haidari

Harold Alderman

Kefyalew Alene

Jason Ali

Mostafa Ali

David Allan

Gabriel Alobo

Maria C Alonso Blanco

Ziad Aloukati

Abdullah Alqarihi

Mekibib Altaye

Miriam Alvarado

Kjell Alving

Israel Amirav

Karin Amrein

Senthil Amudhan

Juan-Manuel Anaya

Peter Anderson

Richard Anderson

Antonio Andreacchio

Kristin Andrejko

Carla Andreucci

Ranjit Mohan Anjana

Daniela Anker

Djillali Annane

Botond Antal

Arnoldo Aquino-Gálvez

S M Yasir Arafat

Christos Aravidis

Gregory Armstrong

Louis Aronne

Monika Arora

Marianna Arvanitakis

Nidal A Asaad

Takashi Asai

Diane A I Ashiru-Oredope

Lisham Ashrafioun

Elizabeth Asztalos

Ilaria Attili

Jean-François Augusto

Angelo Avogaro

Oyekoya Ayonrinde

Arun A Azad

Sina Azadnajafabad

Domenico Azzolino

Karan Babbar

Abraham Babu

James Badenoch

Rahim Badrfam

Jared Baeten

Stéphanie Baggio

Luis Bahamondes

Silver Bahendeka

Shasha Bai

Stephen Bain

Jeffrey Bakal

Laura Baker

Mark Baker

Richard Baker

Timothy Baker

Katherine Balantekin

S Ballaz

Almas Bandeali

Erika Bandini

Ananda Bandyopadhyay

Dhrubajyoti Bandyopadhyay

Debasish Banerjee

Agarwal Banwari

Chunhui Bao

Yanping Bao

Jacob Bar

Lindley Barbee

Joseph Barbi

John L Barbur

Anthony Barnett

Aluisio Barros

Elena Bartoloni

Chamara Basnayake

Marco Basset

Philippe R Bauer

Thomas Bauer

Sina Bavari

Richard Beasley

Lisa Bebell

Kendra Becker

Lance B Becker

Jürg Beer

Samira Bell

Luca Saverio Belli

Louise Bennet

Kaustav Bera

Marge Berer

Laura Bergantini

James Berkley

Ivan Berlin

Stéphane Besançon

Daniel Bessesen

Ashish Bhalla

Deevia Bhana

Sunil Bhandari

Vineet Bhandari

Aditi Bhargava

Debdutta Bhattacharya

Zulfiqar Bhutta

Gashaw Andargie Biks

Franck Billmann

Thomas Bilterys

Giuseppe Biondi-Zoccai

Federico Biscetti

Gregory Bisson

Tuhin Biswas

Melissa Bloomer

Paula Boaventura

T Sonia Boender

David Boettiger

Narikazu Boku

Magnus Boman

Maryline Bonnet

Claudio Borghi

Judith Borhouts

Andrea Botticelli

Jean-Pierre Bouchard

Barbara Boucher

Roger Bouillon

Katherine Bowman

Ben Bradley

Westyn Branch-Elliman

Clinton Brawner

George Bray

João Breda

Hermann Brenner

Thorsten Brenner

Stéphanie Breukink

Elizabeth Brickley

Gabriel Broocks

Adrian Brown

Wendy Brown

Jens Ivar Brox

Valentina Bruno

E J Bryant

Pamela Buchwald

Rachel Buckley

Dario Buioni

Luke Buizen

Adomas Bunevicius

Danilo Buonsenso

Éilish A Burke

Paul Burton

Helen Burton Murray

Matthew Bush

Jorie Butler

Sara Buttery

Nancy Byatt

Richard Byng

Michal Byra

Giuseppe Cabibbo

Dorina Cadar

Florentino Luciano Caetano dos Santos

Guoxiang Cai

Miao Cai

Qiuyin Cai

Jan Calissendorff

Xavier Calvet

Andrea Calvo

Razieh Cameron

Raffaela Campana

William Camu

Carlos Cañas

Bin Cao

Jacqueline Capeau

Luisa Caponecchia

Gabriella Captur

Maria Luce Caputo

Nicola Caranci

Waldemar Carlo

Rodrigo Carrillo-Larco

Ben Carter

Brooks Cash

Jorge Castillo

Jessica Cataldi

Vincenzo Catrambone

Thierry Caus

Valeria Cavalcanti Rolla

Luigi Cavanna

Arthur Caye

Guillermo Cecchi

German Cediel

Luigi Celio

Gilda Cennamo

Sankha Chakrabarti

Dipshikha Chakravortty

Shiao-Yng Chan

Pankaj Chandak

Wing Chung Chang

Keith Chappell

Umesh S Charantimath

Georgia Chaseling

Maria Chatzimina

Nazia Chaudhuri

Sanjay Chawla

Xiaofang Che

Michael Chee

Emerson Chen

Guanhua Chen

Hao Chen

Huaiyong Chen

Kuan-Fu Chen

Lawrence Chen

Lian-Yu Chen

Long Chen

Mu-Hong Chen

Xiang-Sheng Chen

Yong-Ping Chen

Joyce Y Cheng

Xiaoguang Cheng

Felix Chilunga

Adriano Chiò

Shiv Chitturi

Gabriel Chodick

Hayoung Choi

Sherry Chou

Sherry Chou

Elaine Chow

Parul Christian

Ole Christiansen

Felix Chun

Fortunato Ciardiello

Ethan Cicero

Marie-Annick Clavel

James Cleary

Francesco Clemenza

Tammy Clifford

David H Cloud

Javier Cobo

Bruce Cogill

Cheryl Cohen

Jérémie Cohen

Simon Conroy

Matthew Cooperberg

Andrew Copas

Rita Cordeiro

José A Cordero-Ampuero

Rosie Cornish

Ignacio Correa-Velez

Maria Laura Costa

Elizabeth Cotter

Mark Cotton

Frances Cowan

Darren Cowzer

Mark Cox

Vivian Cox

Tamaryn Crankshaw

Ioana Cristea

Julio Croda

Randy Cron

Fuqiang Cui

Xinwu Cui

Kathryn M Curtis

Stefania Cuzzubbo

JP Dadhich

Themistoklis Dagklis

Min Dai

Niamh Daly

Paolo D'Amico

Rakhi Dandona

Scott Dankel

Jamel Daoud

Kassa Darge

Lincoln Darla

Gary Darmstadt

Ara Darzi

Theodore Dassios

Mehul Dattani

Patrick Davies

Adrian Davis

Alan Kooi Davis

Andrew Davis

Thomas Day

Carlos De Angelis

Irith De Baetselier

Ian H de Boer

Giovanni de Girolamo

Thibault de la Motte Rouge

Daniele De Luca

Caroline de Schacht

Thushan de Silva

Mariana de Souza

Chaicharn Deerochanawong

Isabelle Dehaene

Maria Del Ben

Huseyin Demirbilek

Anna Dencker

Annick Desjardins

Kristen DeStigter

John W Devlin

Arnab Dey

Hiba Dhanani

Kumud Dhital

Mauro Di Bari

Luigi Di Filippo

Pierpaolo Di Micco

Andreas Diacon

Gerard Dijkstra

Joakim Dillner

Yingying Ding

Diana Maria DiNitto

Nomita Divi

Vy Doan

Matthew Dodd

Peter Dodd

Hiten Dodhia

Torsten Doenst

Di Dong

Sabrina Donzelli

Kelly Doran

Nicholas Douville

Alex Dregan

Franchek M Drobnic

Mulong Du

Xianglin Du

Shao-Bin Duan

Bruce Duncan

Tara DuPont

Andre Duraes

Sugandha Dureja

Christian Dürsteler

Geoffrey Dusheiko

Alok Kumar Dwivedi

Laura Dwyer-Lindgren

Ava Easton

Alanna Ebigbo

Bernard Ebruke

Julien Edeline

Katie A Edwards

Hidetaka Eguchi

Harald Ehrhardt

Eleni Elia

Kathrin Eller

Alicia Ellis

Nader El-Mallawany

Margit Endler

Kathrin M Engel

Mindy Engevik

Gabriele Escherich

Christopher I Esezobor

Jordi Espadaler Mazo

Lise Estcourt

Anthony Etyang

Paolo Eusebi

Lisbeth Evered

Denise Eygendaal

Fabio Facchinetti

Ahmad Taufik Fadillah Zainal

Faraz Faghri

Carole Fakhry

Melissa Falcao

Ayodele Faleye

Olusola Faluyi

Dongsheng Fan

Junning Fan

Hai Fang

Li-Qun Fang

Meiyu Fang

Eric Faragher

Paddy Farrington

Forough Farrokhyar

Christine Faustino

Jens Fedder

Henry Fein

Charles Feldman

Lori Feldman-Winter

Hua Feng

Claudio Fenizia

Seyed-Mohammad Fereshtehnejad

Rebeca Fernandez

Clarissa Ferrari

Pietro Manuel Ferraro

Massimo Filippi

Carsten Finke

Florian Fischer

Abigail Fisher

Emma Fisher

Kathryn C Fitzgerald

Robert Fleischmann

Jennifer Flemming

Stuart Flint

David Flood

Daniele Focosi

Nathan Ford

Paa Forson

Lauren A Fowler

Michael Fox

Alba Fresco-Taboada

Florian Freudenberg

Louisa Friedrichs

Allison Frost

Edouard Fu

Zhiwen Fu

Marat Fudim

Nobukazu Fujimoto

Yukiyoshi Fujita

Anne Sofie Furberg

Jennifer Furin

Junji Furuse

Frances Game

Milena Gandy

Nirmal Ganguly

Ke Gao

Xin Gao

Claus Garbe

Pablo García de Frutos

Oscar Garcia-Algar

Kathryn Gardner

Coral Gartner

W Timothy Garvey

Jerzy Gąsowski

Sivaporn Gatechompol

Suzanne Geerlings

Nicolas Gendron

Pietro Gentile

Marco Geraci

Patrick Gerardin

Waleed Ghanima

Davide Ghinolfi

Noor Giasuddin

Andrew Gibbs

Chelsia Gillis

Philippe Girard

Sylvie Girard

Corrado Girmenia

Stanton Glantz

Nancy Glass

Norbert N Gleicher

Se-il Go

Brian Godman

Christian Gold

Jianfeng Gong

Davide Gori

Andrew Gorringe

Robert Gottlieb

Anders Gottsäter

Jiangtao Gou

Lisa J Gould

Antonios Gounaris Gounaris

Tadeja Gračner

Alessandro Granito

William Grant

Kelsey Grantham

Stuart Greenstein

Brian Greenwood

Ronni Michelle Greenwood

Edward Gregg

Guillaume Grenet

Chloe Grimmett

Giuseppe Grosso

Vanessa Grubbs

Henning Gruell

Susanne Grylka-Baeschlin

Jérémie Guedj

Bertrand guidet

Moti Gulersen

Martin Gulliford

Boya Guo

Zhiyong Guo

Rajeev Gupta

Kapil Gururangan

Steve Gutreuter

Bishal Gyawali

Edina Hadziselimovic

Christian Hakulinen

Pranabashis Haldar

Brian Hall

Wayne Hall

Pedro Hallal

Xiudi Han

Ziqiang Han

Peter Hanlon

Colleen Hanrahan

Claudia Hanson

James Harding

Simon Harding

Sioban Harlow

Diane Harper

Karen Harrison Dening

Prue Hart

Miriam Hartmann

Jan Hartvigsen

Md Jahidul Hasan

Cesare Hassan

Holger Hauch

Sarah Hawkes

Shabina Hayat

Na He

Qiushui He

Yazhou He

Yulei He

Genevieve Healy

Eric Heinz

Raimund Helbok

Christian Hellum

Julie Hennegan

Georges Herbein

Gonzalo Hernandez

Martin Herrmann

Christian Heumann

Michihiro Hide

Alisa Higgins

Rosemary Higgins

Andrew Hill

Hirofumi Hirakawa

Jane Hirst

Van Thuan Hoang

Rebecca Hock

Markus Hoffmann

Hans Hogerzeil

Trygve Holmøy

Paul Holtzheimer

Caroline Homer

Yves Horsmans

Susan Horton

Mariko Hosozawa

Zhiyong Hou

Chih-Wei Hsu

Derek Y Hsu

Xiaowen Hu

Fei Huang

Judith Nicky Hudson

David Hui

Edwin Hui

Keith Humphreys

Ivan F N Hung

Jacqueline Hunter

Syed Ali Husain

Bing-Fang Hwang

Daniel Borch Ibsen

Romain Icick

Jessica Illuzzi

Ana-Maria Iosif

Fahad Iqbal

Alex Iranzo

Sebastian Irudaya Rajan

Masao Iwagami

Yuishin Izumi

Matthieu Jabaudon

Georges Jabbour

Abbygail Jaccard

Helen Jack

Louise J Jackson

Jeremy Jacobs

Michel Jadoul

Elaine Jaffe

Yasmin Jahan

Konrad Jankowski

Rohan Jayasuriya

Rikke B Jensen

Lutz Jermutus

Susanne Jernelöv

Vivekanand Jha

Peng Jia

Xiangdong Jian

Meng Jiang

Yinzi Jin

Odd Erik Johansen

Douglas Johnson

James Johnston

Mira Johri

Jost Jonas

Paul Wyatt Jones

Linus Jonsson

John Joska

Boyoung Joung

Michael Joyner

Steven Julious

Christian Jung

Heewon Jung

Jennifer Juno

Markus Juonala

Prapan Jutavijittum

Rawen Kader

Yoshihiro Kakeji

Gagan Kalra

Sivesh Kamarajah

Yoichiro Kamatani

Roopa Kanakatti Shankar

Sunanda Kane

Yoshihiro Kanemitsu

Bharat Kantharia

Meinolf Karthaus

Markos Kashiouris

Tyler Kaster

David Katzka

Yoshiharu Kawaguchi

Yoshitaka Kawai

Mamoru Kawakami

Daiji Kawanami

Akihito Kawazoe

Ashish KC

Yang Ke

Karen Keddy

Jeremy Keenan

Danya E Keene

Kelly James Kelleher

Mark Kelson

Zoe Kelson

Rick Kennedy

Stephen Kerr

Ali Keshavarzian

Shelli Kesler

Yousef Khader

Nikolaï Khaltaev

Naushad Khan

Ali Khashan

Samuel Kidane

Rachel Kidman

Christian Kieling

Kim Kiely

Dimitrios Kikidis

Shaun Kilty

Jung-Sun Kim

Song Cheol Kim

Soo-young Kim

Dorien Kimenai

Tomonori Kimura

Hedy Kindler

Ryo Kinoshita

Tyree H Kiser

Amar Kishan

Sarah Kittel-Schneider

Andrew Kitua

J H Kleeff

Claus Klingenberg

Kerstin Klipstein-Grobusch

Paul Knoebl

Richard Kock

Hirofumi Kogure

Kairi Kolves

Anna Kone

Tsuyoshi Konishi

Evangelos Kontopantelis

Konstantinos Kostikas

Adam Kowalczyk

Frederik Kraglund

Georg Kranz

Rathika Krishnasamy

Louise Kuhn

G Anil Kumar

Praveen Kumar

Rakesh Kumar

Shaji Kumar

Vivek Kute

Yury Kuzmichev

Amos Laar

Christian Labenz

Marc LaForce

A Laios

John Lake

Zaher Lakkis

Giovanni Landoni

Giuseppe Lanza

Dennis Lapuente

Heidi Larson

Joseph LaTorre

Wallis Lau

Carl Lavie

Pascal Lavoie

Jeffrey Lazarus

Marzia Lazzerini

Marzia Lazzerini

Carel le Roux

Werner Leber

A Lee

Catherine Lee

Grace J Lee

Matthew Lee

Márcio Lehmann

Lianlian Lei

Amanda Leiter

Maria Lembo

Joseph M Letourneau

Wai Leung

Marcel Levi

Mindy Levin

Stephan Lewandowsky

Dan Lewer

Leslie Edward Simon Lewis

Helena Lewis-Smith

Huiping Li

Jian Li

Jianyong Li

Jingxin Li

Lihong Li

Liming Li

Lin Li

Mian Li

Xiaobo Li

Yi-Gang Li

Yongqin Li

Yuanyuan Li

Zixiao Li

Jean Li-Kim-Moy

Christina Lill

Renly Lim

Sheng Yang Lim

Jimmy Limdi

Pingting Lin

Jan Lindeman

Anna Karin Lindroos

Michael Lindsey

Paul Lips

Gianluca Lista

Kristin Litzelman

Chen Liu

Chiachyi Liu

Cun-Zhi Liu

Jing Liu

Xiaoqin Liu

Per Ljungman

Josep M Llibre

Brian Lo

Kenneth Lo

Wei-Lun Lo

Maya Lodish

Marie Löf

Pedro Lopes de Melo

David Loughrey

Liming Lu

Yihan Lu

Nuttha Lumlertgul

Lixia Luo

Yosu Luque

Deirdre J Lyell

Georgios Lyratzopoulos

Lina Ma

Xuelei Ma

Andrew Maas

Teresa Macarulla

Jacqui Macdonald

José Machado

Graeme MacLaren

Graeme MacLennan

Catriona Ida Macleod

Peter MacPherson

Eric Macy

Ernesto Maddaloni

Sten Madsbad

Markus Maeurer

Simin Mahinrad

Morteza Mahmoudi

Mary Mahy

Govind Kumar Makharia

Thokozile Malaba

Kenneth Maleta

Andrew Mallett

Monica Malta

Mohammed Mamun

V Manchaiah

Vinaya Manchaiah

Swapna Mandal

Mattias Mandorfer

Arduino Mangoni

David M Mannino

Mohammad Ali Mansournia

Gelsomina Mansueto

Maria Maraki

Christian Yahya Mardin

Maria Fatima Marinho de Souza

Gian Luigi Marseglia

Adrian Martineau

Saul Martinez-Horta

Rosa María Martín-Mateos

Neil Martinson

Jane Masoli

Colin Masters

Nestoras Mathioudakis

Ignacio Matos

Amy Matser

Elisabet Mattsson

Sébastien Maury

Sophie I Mavrogeni

Mohsen Mazidi

Giulia Mazzaschi

Sam Mbulaiteye

Stephen McCall

Kimberly J McClure Brenchley

Valerie Mccormack

Benjamin Joseph James McCormick

Peter Aiden McCormick

Vickie McDonald

Nikolaus McFarland

John McGrath

Gerald McGwin

Karen A McLeod

Richard McManus

Iain McNeish

Zoe McQuilten

Jakob Mechler

Francisco Medrano

Dieter Melchart

Erik Melén

Sarra Melki

Walter Mendoza

Xiangming Meng

Sylvain Meylan

Frederic Michard

Lori Michau

Philippa Middleton

Wolfgang Miehsler

Jochen O Mierau

Sarah Miller

John Mills

Geltrude Mingrone

Philip Minor

Antonella Minutolo

Alex Miras

Laurent Misery

Kris Mogensen

Viswanathan Mohan

Ben Willem Mol

John Mollon

Elizabeth Molyneux

Paul Monach

Gemma Moncunill

Charles Mondo

Maciej Monedeiro-Milanowski

Deborah Money

Paolo Montaldo

Maristela Monteiro

Giovanni Monteleone

Alexander Montoye

Simon Mooijaart

Il Joon Moon

Anita Moon-Grady

Donald C Moore

Thierry Mora

Caitlin Moran

Alvaro Moreira

Ian Morgan

Satoru Morimoto

Paul Mork

Patrick Morrison

Kolawole Samuel Mosaku

Ellen Moseholm

Susan Moug

Aya Mousa

Mariam Mousa

Eli Muchtar

Judith Mueller

Julia Mueller

Elliott Mufson

Mirthe Muilwijk

Debabrata Mukherjee

Deborah Mukherji

Humphrey Mulenga

Praveen V Mummaneni

Srinivas Murthy

Alfred Musekiwa

Cristina Mussini

Mathieu Nacher

Cedric Nadeau

Mohsen Naghavi

Suresh Narayanan Nair

Yousuke Nakai

Fred Nalugoda

Angela Narayan

Loretta J Nastoupil

Ruvandhi Nathavitharana

David Naumann

Rebecca H Neiberg

Ujjwal Neogi

Peter Nestor

Josef Neu

Daniel Neureiter

Adriana Nevarez Flores

Robert Newton

Moses Ngari

Jean Paul Nǵuyen

Stephen J Nicholls

Brooke Nichols

Ole Haagen Nielsen

Hendrik Niemarkt

Louis Niessen

Juan Nieto

Kristian Bernhard Nilsen

Peter Nilsson

Monica Paschoal Nogueira

Masanori Nojima

Nurulamin Noor

Liv Nordestgaard

Anna Norhammar

John Norrie

Justine Norton

Christiana Nöstlinger

Seyed Mehdi Nouraie

Mahdad Noursadeghi

Rodrigo Núñez-Cortés

David Nutt

Amy L Nyman

Hendrik Odendaal

Kyle O'Donnell

Jonathan Adedayo Odukoya

Sabine Oertelt-Prigione

Ayodele Ogunleye

Seb Oiwoh

Aikou Okamoto

Naohiro Okano

Nisreen Okba

Joseph Okebe

Ikechi Okpechi

Emmanuel Okpetu

Marcel Olde Rikkert

Mark Oldham

Hélder P Oliveira

Charles Opondo

Esteban Ortiz-Prado

Sophie Orton

Raoul Orvieto

M Osti

Giovanni Ostuzzi

Wim Otte

Quchang Ouyang

Pavel Ovseiko

Alex Pagnozzi

Amir Haji Agha Pakpour

Joseph Palamar

Vanessa Palzes

Prashant Panda

Gaurav Pandey

Manish Pareek

Jean-Jacques Parienti

Simone Parisi

Daniel Park

Sanghee Park

Tony Parks

Christopher Parrish

Francesco Passiglia

Ami Patel

Dimitrios Patoulias

Mary Ellen Pavone

Timothy Pawlik

Lillian Pazvakawambwa

Robert Peach

Markus Peck-Radosavljevic

Kristina L Penniston

Richard Penson

Manuel Pereira

Jordi Pérez-Tur

Susan Persky

Amber Peterman

Katherine Peters

Eskild Petersen

Janka Petravic

Mysore Keshavmurthy Phanish

Sean Phelan

Cyriac Abby Philips

Patrick Phillips

Laura Pini

Prisco Piscitelli

Dario Pitocco

Milan Piya

Gaia Po’

Mirza Pojskic

Riccardo Polosa

Johanneke Portielje

Rajendra Pradeepa

Guy Pratt

Robin Prescott

Joan Price

A Matthew Prina

Cheryl Pritlove

Mahbub Prodip

Milo Puhan

Eero Pukkala

Mary E Putt

Lu Qi

Xingshun Qi

Gang Qin

Xianhui Qin

Ying Qin

Zhongmin Qiu

Terence Quinn

Kazem Rahimi

Katri Raikkonen

Satyan M Rajbhandari

Johanna Ralston

Rajiv Raman

Ranjith Ramasamy

Jonas Ranstam

Kilian Rapp

Marion Rapp

Emanuel Raschi

Robert Raschke

Wafaa M Rashed

Nicholas Ravanelli

Eric Ravussin

Jennifer Rayner

Asnat Raziel

Oliver Razum

Candida Rebello

Krishnaveni Reddy

Mettu Reddy

Emily Reeve

Giuseppe Remuzzi

Ravi Retnakaran

Cézane Reuter

Vaneska Reuters

Luis Reyes

Davide Giuseppe Ribaldone

Alan S Rigby

Gregory Riggins

Edward Riley

Alessandro Rizzo

Angela Roberts

Emily Roberts

Xavier Roblin

Bianca Rocca

Patrick Rockenschaub

Gary Rodin

Thomas Roederer

Elizabeth Rogawski McQuade

Alan Rogol

Nigel Rollins

Dora Romaguera

Roman Romero-Ortuno

Loris Roncon

Damien Roosweil

Annika Rosengren

Peter Rossing

Daniele Rossini

Theresa Rossouw

Lionel Rostaing

Alex Rowlands

Thorsten Rudroff

Juan J Russo

Michelle K Ryan

Neil A J Ryan

Ramakrishnan S

Charumathi Sabanayagam

Fernanda Sabatini

Maria Sae-Hau

Marc Saez

Kamal Sahu

Mark S Salzer

Frederic Sampedro

Fabian Sanchis-Gomar

Jannie Sand

Yingying Sang

Sophia Nicole Sangiorgio

Maíra Santana

Damian Santomauro

Gaetano Santulli

Fred Sarfo

Sabine Sarnacki

Naveed Sattar

Karinna Saxby

Andrew J Saxon

Matthew Schabath

Andre Scheen

Gerit Holger Schernthaner

Alexander Schwed

Andrew Scott

Annalisa Sebastianelli

Shahjada Selim

Gianluca Serafini

Sanjay Sethi

Anoop Shah

Xianwen Shang

Ron Shapiro

William Sharfman

Pankaj Sharma

Shahzad Shaukat

Kayleigh Sheen

Yahya Shehabi

Fadi Shehadeh

Jeanne Shen

Ruihua Shi

Si Shi

Yuan Shi

Yuan Shi

Yuyan Shi

Yugo Shibagaki

Kazuki Shimizu

Kohei Shitara

Dave Shukla

Lawrence Shulman

Karolina Sikorska

João Silva Jr

Morton Silverman

John Simpson

Donald Sin

Gurvinder Pal Singh

Pratik Sinha

Mark Sinyor

Sanjay Sisodiya

Alexandra R Sitarik

Piotr Skarzynski

Hadi Skouri

Lone Skov

Katherine Sleeman

Vyacheslav S Smirnov

Jonathan Smith

Kim Smits

Kristi J Smock

Radoslaw Sobota

Yehoshua Socol

Paolo Soda

Renata Solimini

Jin Woo Song

Ranran Song

Guru Sonpavde

Silvia Sookoian

Cynthia So-Osman

Massimiliano Sorbello

Kjetil Soreide

Elias Sotirchos

Francesca Spada

Jean-Philippe Spano

Elena Spinelli

Laura M Spring

Shobhit Srivasta

Aradhana Srivastava

Tomasina Stacey

Jon Stamford

Joseph Standing

Justin Stebbing

Ambra Stefani

Coen Stehouwer

Mark Stein

Ashleigh Stewart

Nelia Steyn

Shana Stites

Martin Stockler

Timo Strandberg

Pasquale Striano

Ulf Strömberg

Karien Stronks

Diansan Su

Ashwin Subramaniam

S V Subramanian

Masaya Sugiyama

Giorgia Sulis

Ying Sun

Aditya Surapaneni

Paweena Susantitaphong

Mark Sussman

Maria Svensson

Fabio Silvio Taccone

Alayna Tackett

Marina Tadolini

Nicholas Talley

Martin Tammemagi

Atsushi Tanaka

Junko Tanaka

Reiko J Tanaka

Kuo Tung Tang

Pieter Tanis

Robert Tanz

Lionel Tastet

Leah Taylor

Cüneyt Tayman

Helena Teede

Marleen Temmerman

Nikolaos Tentolouris

Charlotte Teunissen

Alesha Thai

Gita Thanarajasingam

Sudhin Thayyil

Eric Peter Thelin

Vincent Thibault

Kim Thomas

Graham Thornicroft

Jurgen Tijms

Dirk Timmerman

Kelvin To

Ingo Todt

Yosuke Togashi

Masakazu Toi

Kihara Tomomi

Øivind Fredvik Grytten Torkildsen

Hidenori Toyoda

Kevin K Tremper

Adam Trickey

Manjari Tripathi

Rebecca Troisi

Stella Trompet

Jennifer Tsai

Ioannis Tsakiridis

Tuo-Yen Tseng

Georgios Tsivgoulis

Lungiswa Tsolekile

Elena Tsourdi

Shoji Tsuji

Jean-Jacques Tuech

Samra Turajlic

Aydın Türkmen

Carolyn Turvey

Gilad Twig

Chryssoula Tzialla

Riaz Uddin

Caroline Um

Yutaka Umemura

Juan Jose Uriarte

Orhan Uzun

Luissa Vahedi

Tracy Vaillancourt

Jonathan Valabhji

Thomas Valley

Anneloes van Baar

Tjip van der Werf

Susan van Dieren

Michiel Van Dijk

Erwin van Geenen

F J van Kemenade

Michel van Putten

Simon Vandekar

Kanika Vanshylla

Paola Varleta

Priyathama Vellanki

Lionne Venderbos

Alison Venn

Amit Verma

Gómez-Lobo Veronica

Anne Vertigan

Timo Vesikari

Peter Vickerman

Josep Vidal

Jean-Louis Vincent

Gianni Virgili

Carmine Vitale

Vasiliy Vlassov

Carmen Vleggeert-Lankamp

Rozemarijn Vliegenthart

Birgit Vogel

Wieland Voigt

Theo Vos

Merryn Voysey

Miroslav Vujasinovic

Stela Vujosevic

Alisha Wade

Hans Werner Wahl

Jaques Waisberg

Melanie M Wall

Kristin Wallace

Jo Waller

Matthew G Wallis

Robinson Wammanda

Luke Wander

Duolao Wang

Fangzheng Wang

Luowei Wang

Zhenning Wang

Zhongfang Wang

Zachary Ward

Heather Wardle

Sean Wasserman

Roger Webb

Michael A Weber

Malte Wehmeyer

Paul Weiss

Alice Welbourn

Bridget Weller

Chi Pang Wen

Thomas Wenzel

André Werneck

Robert Wesolowski

Donald Wesson

Christoph Westphalen

Lisa Weyandt

Kate White

Sarah White

Annelies Wilder-Smith

Donald Willcox

Hywel Williams

Janet Williams

Nick Williams

Jason Willis

Peter Winocour

Alan Winston

Jean Winter

Andre Wirries

Andrea Wirtz

Max Wiznitzer

Paul Wong

Jean Woo

Erica Wood

Shannon Wood

Mark Woodward

Chih-Hsing Wu

Dan Wu

Lianlian Wu

Maddalena Wu

Yi-Long Wu

Bo Xi

Chunjie Xiang

Dan Xiao

Beibei Xu

Huichun Xu

Weili Xu

Xianghuai Xu

Pragya Yadav

Ravi Yadav

Reza Yaesoubi

Lydia Yahia-Cherif

Hiroki Yamaue

Chingfen Yang

Jun Yang

Junlin Yang

Lu Yang

Xiaozhong Yang

Xueying Yang

Yang Yang

Leonard Yeo

Delenasaw Yewhalaw

Peng Yin

Terry Yip

Koffi Yovo

Burak Yulug

Yoshito Zamami

Atefeh Zandifar

Xiao Zang

Rebecca Zash

Boris Zevin

Fengqing Zhang

Hui Zhang

Meng Zhang

Min Zhang

She Zhang

Yaxiong Zhang

Zhongheng Zhang

Binghao Zhao

Fengmin Zhao

Jing Hua Zhao

Qi Zhao

Shihua Zhao

Chunlei Zheng

Ming-Hua Zheng

Jian-Xin Zhou

Xiaoyun Zhou

Yunping Zhou

Zhou Zhou

Bin Zhu

Feng-Cai Zhu

Huaijun Zhu

Yifan Zhu

Zhengbao Zhu

Hongqing Zhuang

Thomas Zilli

Stephan Zipfel

Paolo Zucali

